# Vitamin D Deficiency Is Associated with Glycometabolic Changes in Nondiabetic Patients with Arterial Hypertension

**DOI:** 10.3390/nu14020311

**Published:** 2022-01-12

**Authors:** Gabriele Brosolo, Andrea Da Porto, Luca Bulfone, Laura Scandolin, Antonio Vacca, Nicole Bertin, Cinzia Vivarelli, Leonardo A. Sechi, Cristiana Catena

**Affiliations:** Department of Medicine, Clinica Medica, University of Udine, 33100 Udine, Italy; gabriele.brosolo@uniud.it (G.B.); andrea.daporto@uniud.it (A.D.P.); luca.bulfone1@gmail.com (L.B.); scandolin.laura@spes.it (L.S.); antonio.vacca94@gmail.com (A.V.); nicole.bertin@uniud.it (N.B.); cinzia.vivarelli@asufc.sanita.fvg.it (C.V.); sechi@uniud.it (L.A.S.)

**Keywords:** glucose tolerance, hypertension, insulin resistance, parathyroid hormone, vitamin D

## Abstract

Recent evidence indicates that mildly increased fasting and post-oral load blood glucose concentrations contribute to development of organ damage in nondiabetic patients with hypertension. In previous studies, vitamin D deficiency was associated with decreased glucose tolerance. The aim of this study was to examine the relationships between serum 25(OH)D levels and glucose tolerance and insulin sensitivity in hypertension. In 187 nondiabetic essential hypertensive patients free of cardiovascular or renal complications, we measured serum 25-hydroxyvitamin D (25(OH)D) and parathyroid hormone (PTH) and performed a standard oral glucose tolerance test (OGTT). Patients with 25(OH)D deficiency/insufficiency were older and had significantly higher blood pressure, fasting and post-OGTT (G-AUC) glucose levels, post-OGTT insulin (I-AUC), PTH levels, and prevalence of metabolic syndrome than patients with normal serum 25(OH)D. 25(OH)D levels were inversely correlated with age, blood pressure, fasting glucose, G-AUC, triglycerides, and serum calcium and PTH, while no significant relationships were found with body mass index (BMI), fasting insulin, I-AUC, HOMA index, and renal function. In a multivariate regression model, greater G-AUC was associated with lower 25(OH)D levels independently of BMI and seasonal vitamin D variations. Thus, in nondiabetic hypertensive patients, 25(OH)D deficiency/insufficiency could contribute to impaired glucose tolerance without directly affecting insulin sensitivity.

## 1. Introduction

Type 2 diabetes mellitus, impaired fasting glucose, and the associated insulin resistance are well known risk factors for cardiovascular diseases both in the general population and hypertensive patients [1,2]. Moreover, in subjects with normal fasting glucose, an increased glycemic response to an oral glucose load has been shown to be associated with a greater risk of major cardiovascular events [3], supporting the belief that postprandial blood glucose spikes are important contributors to cardiovascular risk [4]. Recent evidence also indicates that in nondiabetic patients with hypertension, even relatively mild increments of fasting and post-oral load blood glucose levels could contribute to the development of subclinical cardiac [5] and vascular [6] damage. Therefore, due to the possible relevance of blood glucose spikes even in normoglycemic subjects, timely identification of factors that might impair glucose metabolism in nondiabetic hypertensive patients could be important for the prevention or reversal of cardiovascular damage.

In addition to the well-known conditions that predispose to glycometabolic changes such as ethnicity, family history of diabetes, overweight-obesity, inappropriate diets, and physical inactivity, new factors have been recently called into play as possible contributors to glucose metabolism impairment. These factors include inflammatory states [7], circulating levels of adipocytokines [8], the host-microbiota composition [9], and the vitamin D status [10]. In a population-based cohort study, higher serum concentrations of 25-hydroxyvitamin D (25(OH)D) were significantly associated with lower fasting serum glucose levels [10]. Moreover, an inverse relationship between serum 25(OH)D levels and both fasting and 2-h blood glucose after an oral glucose load was reported in predominantly vitamin D-deficient overweight/obese subjects [11], supporting the hypothesis of an association between vitamin D deficiency and decreased glucose tolerance.

Vitamin D deficiency is considered a relatively common condition [12] and a growing body of evidence obtained both in the general population [13] and hypertensive patients [14] suggests the existence of a link between decreased 25(OH)D levels and cardiovascular disease. Moreover, in hypertensive patients, low 25(OH)D levels have been independently associated with subclinical cardiac damage [15]. Mechanisms that might explain the link between vitamin D deficiency and cardiovascular disease are still debated, but might include the effects of vitamin D on glucose homeostasis. With these premises, we sought to investigate the relationships between serum 25(OH)D levels and glucose tolerance and insulin sensitivity in a large group of nondiabetic essential hypertensive patients free of cardiovascular or renal complications.

## 2. Materials and Methods

### 2.1. Patients

In a cross-sectional study, we included 187 patients with essential hypertension who were consecutively referred to the hypertension clinic of our university. The patients seen at our institution include subjects with all grades of hypertension who live in north-eastern Italy and represent the hypertensive population in this geographical area [16]. Blood pressure (BP) was measured by an automated device (Omron M6; OMRON Healthcare Co., Kyoto, Japan) using an appropriately sized cuff after each subject had been lying down for 15 min and the average of three readings was recorded [17]. Diagnosis of hypertension was based on BP measurements obtained in at least three separate visits, according to current guidelines [17]. Exclusion criteria were age < 18 years or >80 years, body mass index (BMI) > 35 kg/m^2^, alcohol abuse, secondary hypertensive disease, diabetes mellitus, dairy product free diet, vitamin D supplementations, treatments that might interfere with blood glucose levels, 24-h creatinine clearance (GFR) < 30 mL/min per 1.73 m^2^, and history of acute illness in the last 3 months. Secondary causes of hypertension were ruled out in all patients according to established guidelines [17], as previously reported [16]. Patients were classified as smokers if they had smoked for at least five years, and up to one year before the study. A questionnaire was used to estimate alcohol intake [18]. The study was performed according to the principles of the Declaration of Helsinki and received approval from the local Institutional Review Board. Informed consent was obtained from all patients.

### 2.2. Vitamin D Status Assessment

Serum 25(OH)D was assayed by chemiluminescence (Immunodiagnostic System, Spello, Italy) with a lower detection limit of 3.6 ng/mL and an intra-assay coefficient of variation of 5.5%. Serum PTH was assayed by chemiluminescence (ADVIA Centaur XP Immunoassay Systems, Siemens Healthcare srl, Erlangen, Germany) with a lower detection limit of 2.5 pg/mL and an intra-assay coefficient of variation of 5.2%. According to the Endocrine Society guidelines [19], vitamin D deficiency was defined as a serum 25(OH)D level lower or equal to 20 ng/mL (50 nmol/L), vitamin D insufficiency as a 25(OH)D level between 21 and 29 ng/mL (525–725 nmol/L), and vitamin D normality as a level of 30 ng/mL (726 nmol/L) or more. To evaluate sunlight exposure, leisure outdoor physical activity was estimated by standardized interviews and patients were defined as physically active if they used to practice an outdoor activity at least 3 h a week. To the same purpose, the season of 25(OH)D measurement was considered, and defined as spring (March–May), summer (June–August), autumn (September–November), and winter (December–February).

### 2.3. Glucose Metabolism Assessment

A venous blood sample was drawn without stasis between 8:00 and 9:00 AM after a 12-h fast. Plasma glucose was measured by the glucose-oxidase method. Plasma insulin and C-peptide levels were assayed by radioimmunoassay. The Homeostatic Model Assessment (HOMA) index was calculated as an index of insulin sensitivity from fasting glucose (mmol/L) and insulin (µU/mL) using the formula: ((glucose × insulin)/22.5). In all patients, an oral glucose tolerance test (OGTT) was performed with a standard load (75 g of glucose) and measurement of plasma glucose and insulin at 30, 60, 90, 120 (G-120), and 180 min [20]. The area under the curve for plasma glucose (G-AUC) and insulin (I-AUC) response to the glucose load was calculated by the trapezoidal rule [21]. Patients with diabetes mellitus were excluded and were identified according to current guidelines [22] by measurement of fasting glucose (≥126 mg/dL), glycated hemoglobin (≥6.5%), and 2-h plasma glucose after OGTT (≥200 mg/dL)). Impaired glucose tolerance was defined by a 2-h plasma glucose after OGTT between 140 and 199 mg/dL [22]. Presence of the metabolic syndrome was defined according to consensus statements [23].

### 2.4. Statistical Analysis

Sample size was calculated to provide a statistical power in the detection of a 25% difference in fasting glucose and insulin, G-AUC and I-AUC between patients with normal or deficient-insufficient serum 25(OH)D level with a probability of less than 5%. Values of normally distributed variables are expressed as means ± standard deviation. Variables with skewed distribution are expressed as median and interquartile ranges (IQR) and were analyzed after logarithmic transformation. Student *t* test was used for comparison between two groups and one-way ANOVA was used for comparison among more than two groups. Pearson’s chi-square test was used to compare frequency distributions. All comparisons were adjusted for covariates. The relationship between continuously distributed variables was examined by linear regression analysis, and the correlation was expressed by Pearson’s correlation coefficient. Multivariate regression analysis was performed to identify which variables were independently related to parameters of glucose metabolism that were correlated with serum 25(OH)D levels in the univariate analysis. In this analysis, variables were sequentially entered in a stepwise model according to the strength of the statistical significance obtained in the univariate analysis. Two-tailed probability values of less than 5% were considered to indicate statistical significance. Data analyses were performed using Stata 12.1 (StataCorp LP, College Station, TX, USA).

## 3. Results

A total of 187 patients with essential hypertension (106 men, 81 women; age, 50 ± 13 years) were included in analysis. In total, 80 of 187 patients had never been treated with antihypertensive drugs. Clinical and biochemical characteristics of the study patients are listed in Table 1, where patients are divided into three groups according to the vitamin D status (deficiency, insufficiency, and normality). A serum 25(OH)D levels below the normal range was present in 107 patients (57%), with vitamin D deficiency in 62 patients (33%) and insufficiency in 45 patients (24%). Patients with 25(OH)D level below normal were older and had significantly higher systolic BP, fasting glucose levels, G-120, G-AUC, I-AUC, PTH levels, and prevalence of metabolic syndrome than patients with normal serum 25(OH)D levels. No differences among groups were found in sex, BMI, duration of hypertension, use of antihypertensive drugs, renal function, fasting plasma insulin and C-peptide, HOMA index, plasma levels of cholesterol, triglycerides, and serum calcium and magnesium. Serum 25(OH)D levels were 23.9 (15.0–33.7) ng/mL in spring (*n* = 57), 37.5 (26.0–60.2) ng/mL in summer (*n* = 26), 29.0 (19.0–49.5) ng/mL in autumn (*n* = 46) and 22.7 (15.7–34.0) ng/mL in winter (*n* = 58) (*p* = 0.021). No significant differences in serum 25(OH)D levels were observed between men and women, physically active and sedentary subjects, smokers and nonsmokers, and between patients who were or were not taking antihypertensive drugs. Additionally, no significant differences in 25(OH)D or glycometabolic variables were observed among patients who were taking different types of antihypertensive drugs.

**Figure 1:**
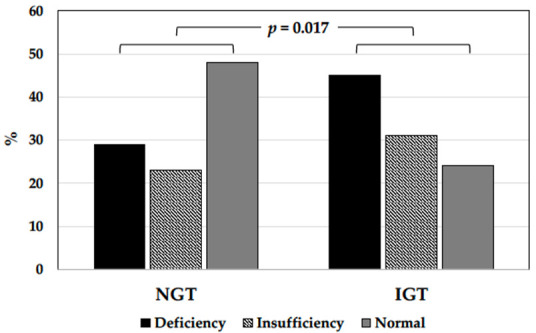
Bar graph showing the prevalence of vitamin D status in hypertensive patients with normal glucose tolerance (NGT) or impaired glucose tolerance (IGT). In nondiabetic hypertensive patients, prevalence of serum 25(OH)D deficiency/insufficiency was significantly higher in patients with impaired than normal glucose tolerance.

Impaired glucose tolerance was present in 45 (24.1%) of 187 hypertensive patients, and the remaining 143 had normal glucose tolerance after the OGTT (Table 2). Patients with impaired glucose tolerance were significantly older, had greater BMI, systolic BP, alcohol intake, and plasma triglycerides, and significantly lower plasma HDL and serum 25(OH)D. As shown in Figure 1, patients with impaired glucose tolerance had significantly higher frequency (*p* = 0.017) of 25(OH)D deficiency/insufficiency than patients with normal glucose tolerance.

**Figure 2:**
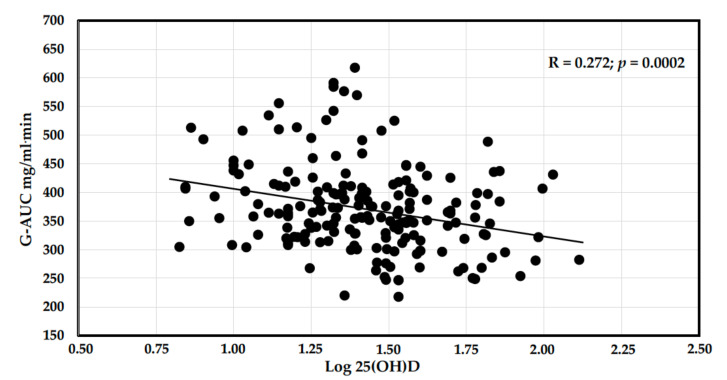
Correlation graph of log 25(OH)D and G-AUC. In nondiabetic hypertensive patients, a highly significant inverse correlation was observed between log transformed serum vitamin D levels and the plasma glucose response curve after an oral glucose tolerance test.

The relationships between 25(OH)D levels and the other study variables were analyzed by univariate linear regression analysis and are shown in Table 3 and Figure 2. This analysis showed that 25(OH)D levels were inversely and significantly correlated with age, systolic BP, fasting glucose and C-peptide, G-120, G-AUC, triglycerides, and serum calcium and PTH, while no significant relationships were found with BMI, diastolic BP, duration of hypertension, alcohol intake, fasting insulin, I-AUC, HOMA index, renal function, and cholesterol levels.

Fasting serum glucose (93 ± 15 vs. 88 ± 10 mg/dL, *p* = 0.003) and G-AUC (384 ± 75 vs. 358 ± 76 mg/dL·min, *p* = 0.021) were significantly higher in men than women and G-AUC was higher in patients who had measurements done in winter-spring than in summer-autumn (383 ± 83 vs. 355 ± 60 mg/dL·min; *p* = 0.008). Univariate analysis of the relationships of glycometabolic variables showed that, in addition to the inverse correlation with 25(OH)D, fasting glucose was significantly and directly correlated with age, BMI, alcohol intake, levels of fasting insulin, C-peptide, triglycerides, cholesterol, and calcium (Table 4). G-AUC was significantly and directly correlated with age, BMI, systolic BP, duration of hypertension, alcohol intake, fasting insulin, C-peptide, I-AUC, PTH, and triglycerides, and G-120 was significantly and directly correlated with age, BMI, systolic BP, duration of hypertension, levels of fasting insulin, C-peptide, I-AUC, and triglycerides.

Multivariate regression analysis was performed including fasting glucose, G-AUC, and G-120, as the dependent variables that were entered as continuous variables (Table 5). Variables identified in the univariate analysis as possible confounders were sequentially entered according to the strength of the association in the univariate analysis. Fasting glucose was independently associated with age, fasting insulin, alcohol intake, and calcium, but not with serum 25(OH)D levels. G-AUC was independently associated with BMI, alcohol intake, I-AUC, serum 25(OH)D levels and measurement performed in summer-autumn. G-120 was independently associated with age, BMI, serum calcium, and 25(OH)D.

## 4. Discussion

We examined the relationships between circulating 25(OH)D and glycometabolic variables in nondiabetic hypertensive patients who were free of cardiovascular and renal complications. The results demonstrate that plasma glucose levels that were measured after a standard oral glucose load are significantly higher in patients with 25(OH)D deficiency/insufficiency than in patients with normal levels of 25(OH)D. Moreover, serum 25(OH)D levels are inversely and independently correlated with post-load blood glucose concentration, but not with fasting and post-load insulin levels or the HOMA index. These findings suggest that in nondiabetic hypertensive patients, low vitamin D levels could contribute to impaired glucose tolerance without directly affecting insulin sensitivity.

The association between vitamin D status and glycometabolic abnormalities was investigated in previous cross-sectional studies. In a Dutch population-based cohort, middle-aged and elderly subjects with adequate 25(OH)D concentrations (≥75 nmol/L) had significantly lower prevalence of elevated fasting glucose than subjects with vitamin D deficiency (<50 nmol/L) [10]. In the Kuopio Study, 25(OH)D concentration was inversely and independently associated with 2-h post-load glucose levels and, after multivariate adjustments, the relative risk for prevalence of type 2 diabetes in decreasing tertiles of serum 25(OH)D was 1.0, 1.26, and 1.44, respectively [24]. In 126 healthy subjects who underwent an OGTT, 25(OH)D levels were inversely correlated with fasting and post-load glucose levels and directly with the insulin sensitivity index [25]. The same significant inverse correlation between circulating 25(OH)D levels and post-load plasma glucose was reported in overweight/obese subjects [11]. In these subjects, however, this relationship was lost after inclusion of the percentage body fat in a multivariate regression model, suggesting that the association between vitamin D and glucose tolerance might be mediated by adiposity. Conversely, studies conducted in American [26] and Asian [27] obese adolescents, many of whom were vitamin D-deficient (<50 nmol/L), did not show any association between vitamin D levels and glycometabolic variables. 

The contribution of the vitamin D status to glucose metabolism changes was also examined in prospective studies. In a German population-based cohort, new onset diabetes was significantly more frequent in women in the lowest quintile of baseline 25(OH)D levels, whereas no association was observed in men [28]. In a subsample of nondiabetic elderly subjects who were included in the Hoorn Study, glucose tolerance test and glycated hemoglobin measurements were performed at baseline and after a median follow-up of 7.5 years [29]. A total of 45 cases of incident diabetes were recorded among 280 study patients, but no significant association was observed with baseline 25(OH)D. In this study, baseline 25(OH)D was significantly and inversely associated with follow-up glycated hemoglobin, although, even in this case, the association disappeared after adjustment for BMI. In line with these results, administration of vitamin D supplements (2800 IU/day) for 8 weeks to 185 subjects with 25(OH)D concentrations lower than 30 ng/mL led to significant reduction of glycated hemoglobin in comparison to placebo-treated controls [30]. In a pilot study of obese subjects of Asian ethnicity, vitamin D supplementation (initial bolus of 2500 mg of cholecalciferol followed by 100 mg/day) for 16 weeks significantly improved fasting blood glucose in comparison to placebo, but no changes were observed in insulin sensitivity as assessed by the hyperinsulinemic clamp [31]. Lastly, the effect of vitamin D supplementation on glycemic control and insulin sensitivity in subjects with prediabetes was examined in a meta-analysis of 10 randomized controlled trials [32]. This analysis reported no effect of vitamin D on insulin sensitivity, but showed significant reduction of fasting glucose, glycated hemoglobin, and 2-h post-load plasma glucose levels in subjects with baseline 25(OH) lower than 50 nmol/L. Thus, despite some variability among the findings of all these studies, the vitamin D status seems to have greater relevance for fasting and post-load glucose control than insulin sensitivity. This is fully consistent with the results of the present study, which extend this evidence to hypertensive patients.

Awareness of the possible relationships between the vitamin D status and glycometabolic changes in hypertension is clinically relevant and might contribute to better definition of cardiovascular risk. In fact, reduced vitamin D concentrations are frequently detected in hypertensive patients [33], in whom impaired glucose tolerance is independently associated with cardiac and arterial damage [2,3,4,5,6]. In a cross-sectional study, Sciacqua et al. examined the relationship of 25(OH)D with variables of glucose metabolism in 300 hypertensive patients, 44 of whom had impaired glucose tolerance and 26 had diabetes [34]. Patients with normal glucose tolerance (*n* = 230) were divided according to an arbitrary cut-off of 1-h post-load plasma glucose of 155 mg/dL. Patients with normal glucose tolerance and 1-h post-load glucose of more than 155 mg/dL had significantly lower plasma 25(OH)D, showing an inverse relationship between vitamin D levels and the glucose tolerance status. These results are consistent with the present findings obtained in a highly selected group of hypertensive patients who were extensively characterized and were free of important confounders such as diabetes, cardiovascular, and renal disease. Another important confounder are seasonal variations of vitamin D levels due to changes in sunlight exposure. These variations were considered in our multivariate model, showing that the inverse relationship of 25(OH)D with glucose tolerance is independent of seasonal changes. Moreover, in our patients, the association of vitamin D with glucose tolerance was independent of sex, BMI, outdoor physical activity, alcohol and cigarette consumption, serum magnesium, and lipid parameters.

Possible mechanisms that might explain the reported association between inadequate vitamin D status and impaired glucose tolerance are related with the widespread presence of vitamin D receptors in tissues or, alternatively, to indirect effects that are mediated by changes in calcium and PTH. Vitamin D receptors were demonstrated in pancreatic β-cells [35] and protect these cells from immune responses [36], thus preserving the insulin secretory capacity of pancreatic islets [37]. Additionally, vitamin D increases insulin receptor expression and insulin activation of glucose transport [38], leading to more efficient cellular glucose internalization. These effects of vitamin D partially contrast with the findings of the present and previous studies that have almost consistently reported an association of vitamin D deficiency with glucose intolerance but not insulin resistance. This is why indirect effects of vitamin D could be called into play, including those on PTH and calcium. PTH was demonstrated to decrease glucose uptake in several tissues [39] and PTH concentrations were significantly increased in our vitamin D-deficient patients. Possible contribution of PTH to glucose intolerance is also supported by observation of significant direct correlation of serum PTH levels with G-AUC. Moreover, in this study, G-AUC was directly correlated with calcium concentrations, and previous studies in subjects with impaired glucose tolerance have shown an association with higher levels of serum calcium [40].

This is the first study to investigate the relationship between the vitamin D status and glycometabolic changes in a highly selected group of patients with hypertension. The major strength of this study is its size, inclusion of patients free of diabetes and cardiovascular and renal complications, and inclusion in the analysis of all major confounders that were extensively characterized in these patients. Some important limitations, however, need to be highlighted. First, the cross-sectional design does not permit to definitely establish causality between vitamin D deficiency/insufficiency and glucose intolerance, although the independence and the strength of the association would suggest so. Second, the use of antihypertensive drugs in a considerable proportion of hypertensive patients might have affected the results, although none of these drugs was shown to affect vitamin D levels. Third, the use of a clinical sample might limit the possibility to generalize the findings of this study to the general population. Last, although previous studies have reported a significant association of the vitamin D status and glycometabolic variables with serum magnesium concentration [41,42], we did not observe this association in our patients. Because this association was reported to be particularly relevant in obese subjects, a possible explanation could be related to the relatively low prevalence of obese subjects in our patient sample.

## 5. Conclusions

Due to its elevated prevalence in the general population, arterial hypertension is considered the principal modifiable cardiovascular risk factor. In patients with hypertension, major cardiovascular events are anticipated by subclinical organ damage. Many factors, in addition to high blood pressure, have a role in the development and progression of hypertensive organ damage. Among these factors, mild increments of fasting and post-oral load blood glucose levels are associated with the development of subclinical cardiac and arterial damage. Additionally, inappropriately reduced vitamin D concentrations are frequently detected in patients with arterial hypertension and might contribute to the cardiovascular risk. This study is the first to demonstrate in a highly selected group of nondiabetic and uncomplicated hypertensive patients that low vitamin D levels are independently associated with impaired glucose tolerance.

These findings might have important clinical implications. Because of the strength of the association of glucose intolerance with reduced vitamin D, the measurement of circulating 25(OH)D in all hypertensive patients could be a useful tool for timely identification of changes in glucose metabolism and better stratification of cardiovascular risk. Appropriately designed interventional trials with vitamin D supplementation in hypertensive patients with inadequate vitamin D status will be needed to establish the relevance of these findings in clinical practice.

## Figures and Tables

**Table 1:** Clinical characteristics and biochemical variables of the study patients according to levels of 25(OH) vitamin D.

Variables	All Patients	25(OH)D	25(OH)D	25(OH)D	*p*
	(*n* = 187)	<21 ng/mL	21–29 ng/mL	≥30 ng/mL	
		(*n* = 62)	(*n* = 45)	(*n* = 80)	
Clinical characteristics					
Age, year	50 ± 13	54 ± 12	52 ± 13	47 ± 11	0.001
Males, *n* (%)	106 (57)	29 (52)	27 (63)	44 (54)	0.526
BMI, kg/m^2^	28.0 ± 5.1	28.7 ± 5.6	28.1 ± 4.4	27.5 ± 5.2	0.394
Heart rate, bpm	71 ± 12	74 ± 12	69 ± 13	71 ± 12	0.931
Systolic BP, mm Hg	149 ± 19	155 ± 21	150 ± 20	146 ± 17	0.038
Diastolic BP, mm Hg	93 ± 12	93 ± 12	94 ± 14	93 ± 12	0.931
Duration of hypertension, y	7 ± 9	10 ± 10	7 ± 8	6 ± 8	0.126
Antihypertensive Tx, *n* (%)	107 (57)	36 (64)	26 (60)	43 (53)	0.404
Alcohol intake, g/day	9 ± 19	7 ± 14	14 ± 25	8 ± 17	0.336
Smokers, *n* (%)	44 (23)	15 (27)	11 (26)	17 (21)	0.704
Physically active, *n* (%)	37 (20)	11 (20)	11 (26)	15 (18)	0.638
Metabolic syndrome, *n* (%)	68 (36)	29 (52)	17 (39)	17 (21)	0.001
Season					0.021
Spring, *n* (%)	57 (30)	22 (35)	14 (31)	21 (26)	0.492
Summer, *n* (%)	26 (14)	2 (4)	6 (14)	18 (69)	0.004
Autumn, *n* (%)	46 (25)	15 (27)	8 (19)	23 (50)	0.391
Winter, *n* (%)	58 (31)	22 (30)	17 (35)	19 (35)	0.173
Summer/autumn	72 (38)	17 (27)	14 (31)	41 (51)	0.008
Biochemical variables					
Serum creatinine, mg/dL	0.93 ± 0.23	0.94 ± 0.31	1.01 ± 0.25	0.99 ± 0.26	0.274
GFR, mL/min^.^ 1.73 m^2^	99 ± 26	92 ± 26	101 ± 26	100 ± 26	0.124
Fasting glucose, mg/dL	91 ± 13	92 ± 14	95 ± 18	88 ± 9	0.031
Fasting insulin, µUI/mL	7.5 (4.2–11.8)	9.40 (4.6–13.1)	7.3 (3.7–12.2)	7.0 (4.1–10.0)	0.478
C-peptide, ng/dL	1.89 (1.35–2.63)	1.99 (1.47–2.84)	1.97 (1.40–2.77)	1.74 (1.21–2.49)	0.097
HOMA index	1.64 (0.86–2.68)	1.97 (1.00–1.77)	1.58 (0.76–3.28)	1.51 (0.86–2.29)	0.368
G-AUC, mg/dL·min	373 ± 76	393 ± 68	397 ± 92	348 ± 66	<0.001
G-120, mg/dl	120 ± 45	131 ± 47	132 ± 60	107 ± 29	0.001
I-AUC, µUI/mL·min	133 (91–200)	141 (99–220)	162 (107–242)	114 (87–158)	0.039
Triglycerides, mg/dL	118 ± 66	130 ± 82	120 ± 60	105 ± 52	0.076
Cholesterol, mg/dL	200 ± 41	204 ± 42	197 ± 46	197 ± 40	0.530
HDL cholesterol, mg/dL	56 ± 17	52 ± 14	55 ± 15	59 ± 18	0.049
LDL cholesterol, mg/dL	120 ± 36	128 ± 38	117 ± 38	117 ± 34	0.245
25(OH)D, mg/mL	26.0 (17.9–37.0)	15.0 (11.2–18.0)	24.6 (22.5–26.0)	39.9 (34.0–60.0)	<0.001
1,25(OH)D, pg/mL	81 ± 52	61.4 ± 44.3	65.6 ± 38.3	104.7 ± 53.6	<0.001
PTH, pg/mL	61 (43–76)	67 (59–86)	59 (47–77)	54 (39–70)	<0.001
Calcium, mg/dL	9.2 ± 0.5	9.3 ± 0.4	9.2 ± 0.5	9.1 ± 0.5	0.086
Magnesium, mmol/L	0.85 ± 0.09	0.85 ± 0.10	0.84 ± 0.08	0.85 ± 0.08	0.881

Data are presented as mean ± SD or as median (interquartile range). Comparison between the three groups were done by ANOVA after log transformation for variables with skewed distribution. To convert to international units, multiply glucose by 0.05551 (mmol/L), insulin by 7.175 (pmol/L), C-peptide by 3.021 (nmol/L), and calcium by 0.2495 (mmol/L). Abbreviations: BMI, body mass index; BP, blood pressure; HOMA, homeostatic model assessment; GFR, glomerular filtration rate; G-AUC, area under the curve for plasma glucose after oral glucose load; G-120, plasma glucose level at 120 min after oral glucose load; I-AUC, area under the curve for plasma insulin after oral glucose load; PTH: parathyroid hormone.

**Table 2:** Clinical characteristics and biochemical variables of the study patients according to normal (NGT) or impaired (IGT) glucose tolerance.

Variables	NGT	IGT	*p*
	(*n* = 143)	(*n* = 45)	
Clinical characteristics			
Age, year	49 ± 12	54 ± 12	0.009
Males, *n* (%)	75 (52)	31 (69)	0.077
BMI, kg/m^2^	27.5 ± 5.3	29.8 ± 4.0	0.007
Heart rate, bpm	70 ± 12	73 ± 12	0.275
Systolic BP, mm Hg	148 ± 17	154 ± 18	0.061
Diastolic BP, mm Hg	92 ± 12	95 ± 11	0.210
Duration of hypertension, year	7 ± 8	9 ± 9	0.119
Antihypertensive Tx, *n* (%)	80 (56)	27 (60)	0.631
Alcohol intake, g/day	8 ± 16	14 ± 24	0.061
Smokers, *n* (%)	35 (24)	9 (20)	0.536
Physically active, *n* (%)	28 (20)	9 (20)	0.951
Metabolic syndrome, *n* (%)	39 (27)	29 (64)	<0.001
Biochemical variables			
Serum creatinine, mg/dL	0.92 ± 0.21	0.96 ± 0.27	0.265
GFR, ml/min^.^ 1.73 m^2^	98 ± 26	96 ± 26	0.645
Fasting glucose, mg/dL	88 ± 9	100 ± 18	<0.001
Fasting insulin, µUI/mL	7.0 (4.0–10.6)	11.0 (5.2–14.9)	<0.001
C-peptide, ng/dL	1.85 (1.21–2.49)	2.44 (1.70–2.91)	0.017
HOMA index	1.51 (0.84–2.29)	2.79 (1.34–4.19)	<0.001
G-AUC, mg/dL·min	344 ± 53	464 ± 67	<0.001
G-120, mg/dL	102 ± 21	178 ± 52	<0.001
I-AUC, µUI/mL·min	105 (88–119)	154 (147–193)	<0.001
Triglycerides, mg/dL	113 ± 68	134 ± 60	0.064
Cholesterol, mg/dL	199 ± 41	201 ± 43	0.825
HDL cholesterol, mg/dL	58 ± 17	50 ± 13	0.010
LDL cholesterol, mg/dL	119 ± 36	122 ± 38	0.636
25(OH)D, mg/mL	29.0 (19.0–38.8)	21.0 (14.0–26.8)	<0.001
PTH, pg/mL	60 (42–74)	68 (48–82)	0.569
Calcium, mg/dL	9.1 ± 0.5	9.3 ± 0.4	0.047
Magnesium, mmol/L	0.85 ± 0.08	0.84 ± 0.11	0.507

Data are presented as mean ± SD or as median (interquartile range). Comparison between the three groups were done by ANOVA after log transformation for variables with skewed distribution. To convert to international units, multiply glucose by 0.05551 (mmol/L), insulin by 7.175 (pmol/L), C-peptide by 3.021 (nmol/L), and calcium by 0.2495 (mmol/L). Abbreviations: BMI, body mass index; BP, blood pressure; HOMA, homeostatic model assessment; GFR, glomerular filtration rate; G-AUC, area under the curve for plasma glucose after oral glucose load; G-120, plasma glucose level at 120 min after oral glucose load; I-AUC, area under the curve for plasma insulin after oral glucose load; PTH: parathyroid hormone.

**Table 3:** Relationships between log 25(OH)D levels and other study variables.

Variables	r	*p*	Variables	r	*p*
Age	−0.255	0.001	HOMA index	−0.118	0.116
Body mass index	−0.065	0.377	G-AUC	−0.272	<0.001
Systolic blood pressure	−0.167	0.021	G-120	−0.222	0.002
Diastolic blood pressure	−0.012	0.866	I-AUC	−0.133	0.079
Duration hypertension	−0.117	0.117	Triglycerides	−0.180	0.014
Alcohol intake	−0.048	0.535	Total cholesterol	−0.121	0.100
GFR	0.005	0.942	HDL cholesterol	0.110	0.136
Fasting glucose	−0.215	0.003	LDL cholesterol	−0.111	0.134
Fasting insulin	−0.076	0.306	PTH	−0.310	<0.001
C-peptide	−0.194	0.010	Calcium	−0.168	0.022

Abbreviations: GFR, glomerular filtration rate; HOMA, homeostatic model assessment; G-AUC, area under the curve for plasma glucose after oral glucose load; G-120, plasma glucose level at 120 min after oral glucose load; I-AUC, area under the curve for plasma insulin after oral glucose load; PTH: parathyroid hormone.

**Table 4:** Relationships between parameters of glucose metabolism and other study variables.

	Fasting Glucose		G-AUC		G-120	
	r	*p*	r	*p*	r	*p*
Clinical characteristics						
Age	0.299	<0.001	0.253	<0.001	0.277	<0.001
Body mass index	0.250	0.001	0.279	<0.001	0.231	0.001
Systolic blood pressure	0.101	0.172	0.296	<0.001	0.195	0.008
Diastolic blood pressure	−0.024	0.747	0.101	0.170	0.036	0.623
Duration of hypertension	0.149	0.049	0.240	0.001	0.249	0.001
Alcohol intake	0.265	<0.001	0.301	<0.001	0.122	0.113
Biochemical variables						
GFR	−0.021	0.779	−0.046	0.532	−0.073	0.323
Fasting insulin	0.372	<0.001	0.264	<0.001	0.226	0.002
C-peptide	0.404	<0.001	0.252	0.001	0.239	0.002
I-AUC	0.134	0.078	0.384	<0.001	0.267	<0.001
Triglycerides	0.172	0.019	0.240	0.001	0.191	0.009
Total cholesterol	0.153	0.037	0.127	0.083	0.017	0.822
HDL cholesterol	−0.133	0.073	−0.140	0.058	−0.187	0.011
LDL cholesterol	0.155	0.036	0.099	0.183	0.009	0.903
25(OH)D	−0.215	0.003	−0.267	<0.001	−0.222	0.002
1,25(OH)D	−0.025	0.808	0.008	0.938	−0.100	0.325
PTH	0.125	0.094	0.190	0.011	0.135	0.070
Calcium	0.212	0.004	0.232	0.002	0.093	0.207

Abbreviations: GFR, glomerular filtration rate; I-AUC, area under the curve for plasma insulin after oral glucose load; 25(OH)D, 25-hydroxyvitamin D3; 1,25(OH)D, 1-25 dixydroxyvitamin D3; PTH: parathyroid hormone.

**Table 5:** Multivariate analysis with fasting glucose, G-AUC, and G-120 as dependent variables.

Fasting Glucose			G-AUC			G-120		
	β	*p*		β	*p*		β	*p*
Age	0.229	0.001	Age	0.108	0.145	Age	0.223	0.009
Body mass index	0.126	0.084	Male gender	0.125	0.093	Body mass index	0.165	0.037
Male gender	0.128	0.067	Body mass index	0.161	0.032	Log insulin	0.079	0.218
Alcohol intake	0.202	0.004	Alcohol intake	0.212	0.005	Log 25(OH)D	0.172	0.025
Log insulin	0.271	<0.001	Log I-AUC	0.245	0.001	Log PTH	0.002	0.983
Log 25(OH)D	0.079	0.243	Log 25(OH)D	0.155	0.047	Calcium	0.155	0.029
Calcium	0.261	<0.001	Log PTH	0.018	0.806			
			Summer/Autumn	0.149	0.041			

Abbreviations: I-AUC, area under the curve for plasma insulin after oral glucose load; 25(OH)D, 25-hydroxyvitamin D3; 1,25(OH)D, 1-25 dixydroxyvitamin D3; PTH: parathyroid hormone.

## Data Availability

Not applicable.
